# Increasing access to care through digital health for the Medicaid population: a novel community case study

**DOI:** 10.3389/fdgth.2025.1524590

**Published:** 2025-09-19

**Authors:** Melinda Cooling, Colleen J. Klein, Matthew D. Dalstrom, Roopa Foulger, Jennifer Junis, Jonathan A. Handler

**Affiliations:** ^1^ OSF OnCall Digital Health, OSF HealthCare, Peoria, IL, United States; ^2^ Advanced Practice, OSF HealthCare, Peoria, IL, United States; ^3^ Graduate Program, Saint Anthony College of Nursing, Rockford, IL, United States; ^4^ Digital Innovation Development, OSF HealthCare, Peoria, IL, United States; ^5^ OSF OnCall Digital Health, OSF OnCall Administration, Peoria, IL, United States; ^6^ Innovation, Clinical Intelligence and Advanced Data Lab, OSF HealthCare, Peoria, IL, United States

**Keywords:** digital health, Medicaid, innovation, population health, chronic disease, pregnancy, wellness

## Abstract

There is a growing consensus among healthcare professionals and policymakers that the way healthcare has historically been provided within the United States is insufficient to meet the needs of the population. The incidence and prevalence of many chronic diseases, coupled with the challenges associated with accessing prenatal care, are notable across the country and globally. In response to this problem OSF HealthCare and four federally qualified health centers partnered together to reimagine how health care can be delivered to underserved populations. This case study provides a practical perspective on how care delivery is enhanced, delivered, and improved through use of digital technologies to expand access to care and chronic disease management in the Medicaid population. Through the formation of the Medicaid Innovation Collaborative, which is partially funded by the Illinois Department of Health and Family Services, digital health programs tailored to individual patient needs and supported by remote and in-person digital health navigators (DHNs), are provided with 24/7/365 access to care from a diverse team of healthcare professionals. In this article, we describe the essential program elements, design, and implementation of four novel programs. While developing digital care solutions for adult Medicaid recipients across the state has been challenging, our work illustrates the feasibility of such an endeavor. To date, we have outreached to over 418,037 patients, and enrolled 38,964 in our diverse programs that include, but are not limited to, helping patients managing chronic disease, increasing access to prenatal care, offering support for health literacy and wellness, and screening for the social determinants of health.

## 1 Introduction

Within the United States (U.S.) and globally, individuals who live with the diagnosis of a chronic disease and/or are pregnant have many obstacles to overcome (1), beyond the physical and psychological symptoms they exhibit (2). The COVID-19 pandemic shed light on the social, economic, and community factors for patients who are disproportionately affected, particularly for those who are low-income and/or within Brown and Black communities (1, 3). Suggestions for population-based health care include innovations such as digital health (4), with its use during the pandemic advancing it as a possible solution. However, the full impact and benefits of digital health in chronic disease management and pregnancy are yet to be fully understood, and further research is needed (2, 5, 6). Supporting health prevention has also proven challenging in low-income communities, with a lack of insurance coverage and access to care as additional significant barriers (7, 8). To address the issues of access and cost, Illinois (IL) was one of the earliest states to expand Medicaid coverage after the passage of the Affordable Care Act. While the expansion reduced the uninsured rate from 12.7% in 2013 to 6.6% in 2022 (9), the incidence rate of chronic disease and poor birth outcomes in Illinois has remained stubbornly high (10), suggesting that insurance coverage and traditional healthcare delivery models are insufficient.

### 1.1 Rationale for the proposed innovation

In 2021, IL passed the Healthcare Transformation Collaboratives Public Act 101-655, which allocated funds to develop cross-provider partnerships that address barriers and access to care, increase the stability of healthcare resources, and to reduce the negative impacts of the social determinants of health (SDoH). In response, OSF HealthCare formed partnerships with several federally qualified health centers (FQHCs) to establish the “Medicaid Innovation Collaborative” (MIC). This collaboration was established as a transformative care model designed to address the needs of Medicaid patients and the broader community. In building the care model, evidence-based digital strategies were used to enhance population health, wellness, and management of specific chronic diseases for all residents in the communities served by our healthcare system, working in collaboration with FQHCs. The 5-year MIC includes the following FQHC partners in IL: Heartland Health Services (Peoria), Chestnut Health Systems, Inc. (Bloomington), Eagle View Community Health System, Inc. (Oquawka), and Aunt Martha's Health & Wellness (Danville) (11).

### 1.2 Community context and health services collaboration

OSF HealthCare (OSF) serves more than 1,026,891 patients across a large geography that spans over 500 miles in two states [IL and Michigan (MI)]. Of the households served by our healthcare system, 46% are low-income, and 22% are below the poverty line. Across our operating regions, the population served includes 24.6% of all Black, 15.7% of all Hispanic, 35.3% of all rural, and 29.6% of all urban residents living in the service area. We have the greatest number of Medicaid patients of any health system in the central region. OSF also serves Medicaid patients through its hospitals and clinics in Chicago, northern IL, and Escanaba, MI. Since the initial expansion of Medicaid, enrollment in Illinois remains substantial with 3,438,477 people enrolled in Medicaid as of August 2024 (12), representing approximately 1 in 6 adults aged 19–64 making Illinois the 5th highest state in the U.S. for enrollment (13).

**Figure 1:**
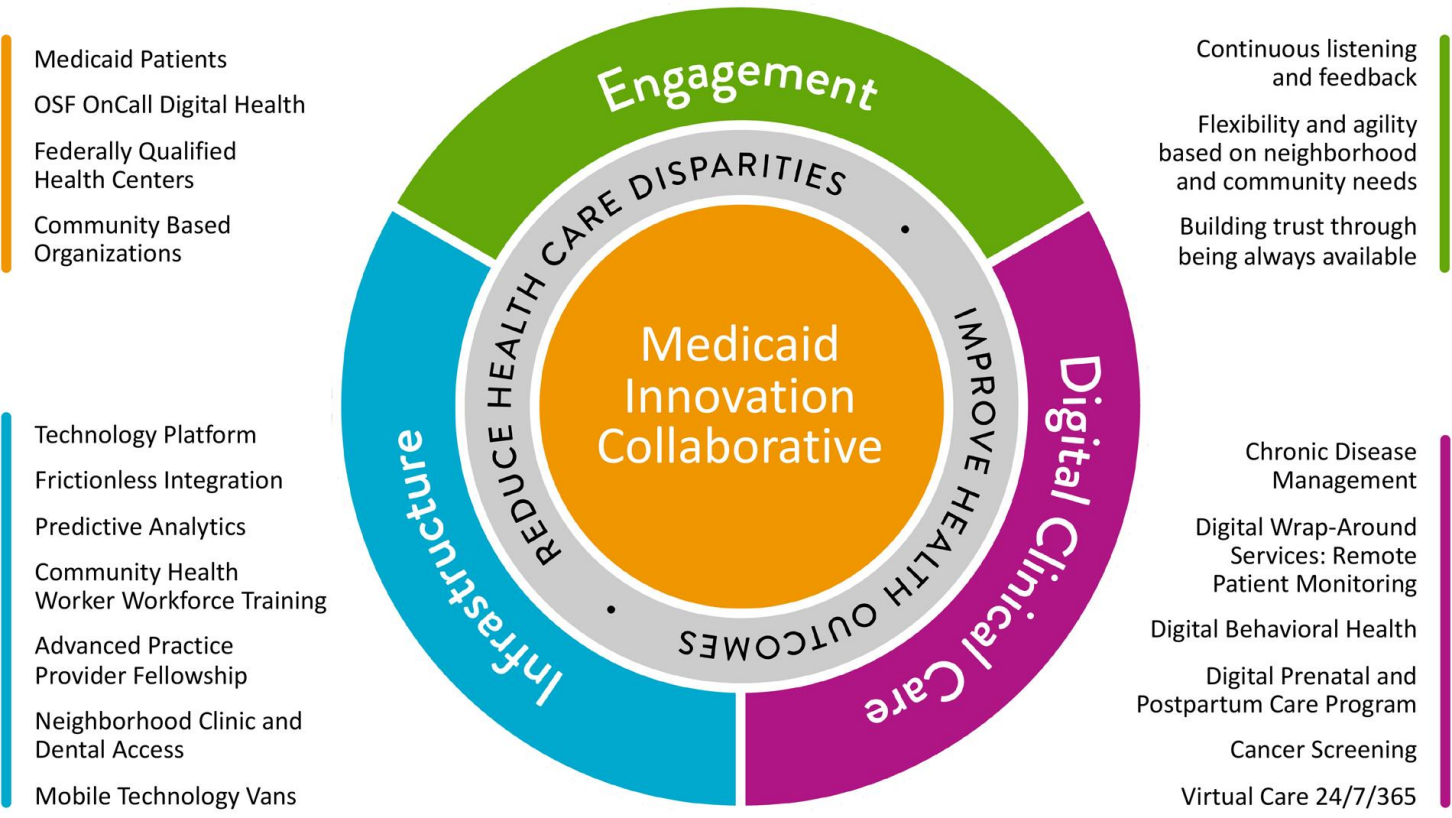
Vision for OSF HealthCare Medicaid Innovation Collaborative (MIC). Conceptual framework depicting the MIC, with listings of key components: partnerships (gold), infrastructure (blue), digital clinical care (magenta) and engagement (green). Image provided by OSF HealthCare.

Our academic partnerships with the University of Illinois (UI) at Chicago and the UI College of Medicine in Peoria (UICOMP), and nonprofit research groups (City Tech Collaborative) (14) served as avenues for gathering data of community perceptions and needs in the pre-funding phase. The most prolific branch of these efforts was the OSF Innovation's Health Equities Action Laboratory (HEAL). In 2017, HEAL began its work to reveal and address health disparities. Their focus was to innovate solutions such as scalable tools, technologies, and interventions, which promoted measurable gains in health equity for our communities (15). A visioning session before the proposal submission was led by OSF HealthCare with FQHC leaders. Figure 1 depicts the MIC conceptual framework guiding this transformative work (11). The MIC partners identified high-priority patient care needs and partnership responsibilities based on existing data, as described above, and from community health needs assessments conducted within OSF and the FQHCs. Governance was established at this time, enabling the MIC program to be implemented immediately after contract finalization in February 2022. In this paper, we will describe the essential elements, results, challenges, current state, and recommendations for possible replication.

## 2 Key programmatic elements

Essential elements of program development and implementation included:

1. designing and deploying multiple innovative programs which include digital solutions (discussion is limited to four digital health programs (to provide sufficient detail),
2. developing a risk algorithm to inform patient appropriateness for outreach related to each program,
3. building a data infrastructure with technology platforms for data curation, data sharing, and interoperability,
4. conducting program evaluations using a pragmatic research approach (16).

### 2.1 Programmatic aspects

#### 2.1.1 Program design and deployment

OSF HealthCare developed a three-tiered telehealth program that aligns patient management strategies with clinical complexity using vendor supported platforms. Individuals with the highest number and severity of chronic diseases, specifically hypertension (HTN), diabetes mellitus (DM), asthma, heart failure (HF), and chronic obstructive pulmonary disease (COPD), are invited to enroll in the high-touch OnCall Advanced Care (OCAC) program, which provides intensive interventions and advanced disease management. Patients with moderate complexity, defined as having at least one of the chronic diseases mentioned above but not qualifying for high-touch program, are offered the medium-touch Remote Patient Monitoring (RPM) program. Those without any of these chronic conditions are enrolled in the low-touch program, Health and Wellness, which focuses on supporting wellness and promoting healthy behaviors. Moreover, pregnant and postpartum women, regardless of their chronic disease status, can enroll in the Pregnancy and Postpartum Support Program (PPSP) starting at 8 weeks of pregnancy and continuing through 6 weeks postpartum. Patients may participate in only one program at a time based on their highest clinical need, but they can transition between programs as their health status changes. Our electronic health record (EHR) clinical data are used to stratify patients with one of five chronic diseases, as well as to identify pregnant and postpartum patients.

A staggered yet aggressive timeline for program implementation occurred during the first 2 years, with continued delivery, refinement, and optimization expected over a total of 5 years. Given the wide variation across FQHCs for technology integration, EHR systems, and internal resources to support digital platforms, this approach was necessary. An extensive listing of deliverables and milestones agreed upon contractually (Supplementary Figure S1), along with “current state” assessments of the FQHCs' infrastructure, served as crucial elements and performance measures of the program's initial phases of development and implementation. OSF began program rollouts using identified OSF patients with Medicaid coverage who met the program criteria, allowing for testing of digital programs and deployment internally before activation and patient recruitment within FQHCs. A systematic approach to assess health disparities was also developed as part of these programs to prioritize patient outreach, improve access to care, and optimize workflow (11).

#### 2.1.2 Developing staffing ratios and program playbooks

Digital programs can often support a large patient population. Nurses, supported by DHNs, commonly handle a much higher ratio of patients than in-person care programs, particularly in the RPM program. Determining clinician-to-patient ratios can be challenging until you have a better understanding of patient needs within a specific program. To help accomplish this during the planning and implementation, executive leaders partnered with operational and clinical leaders. They decided that additional clinical leadership and staff were necessary to provide virtual care delivery options, totaling 15.8 new positions (full-time equivalents) in the first year. Nurses, DHNs, and advanced practice providers (APP) (advanced practice nurses and physician assistants) roles were created and staffed to ensure access to care 24/7/365. Ratios in the first year were appropriate, having learned from our COVID-19 digital end-to-end response (17). A three-tiered approach, supported by technology, enabled care team members to practice within their full scope to test clinician-to-patient ratios. Benchmarking with other healthcare systems working in digital care also helped with initial staffing projections, along with the staggered implementation of FQHCs.

#### 2.1.3 Patient eligibility

To identify patients who are eligible to participate in the MIC programs, we consider those who are 18 years and older, their current insurance status as Medicaid or Medicaid Managed Care, current provider status, and patient relationship to OSF HealthCare as a means to determine their attribution to OSF OnCall, a FQHC, or as a patient with Medicaid without a relationship to either. The programs are free of charge to IL Medicaid recipients who meet the eligibility criteria for each program. Consenting patients are then invited to complete SDoH screening questions with a DHN via phone. Initial questions were based on the evidence-based PRAPARE® Model for FQHCs and included topics such as social integration, safety and domestic violence, financial resource strain, food insecurity, transportation needs, housing needs, and stress (18). A cascade of up to 26 questions are asked to provide additional patient-specific insights at the individual level, allowing for more targeted interventions (11, 16). Our DHNs are then able to refer people to community resources, help them set up clinical appointments at OSF HealthCare, and provide educational resources for patients' questions/requests. Patients are informed that care can only be provided if they are physically present in Illinois due to state of Illinois practice regulations.

### 2.2 Digital health programs

#### 2.2.1 Risk algorithm for patient appropriateness and program determination

As part of our patient identification and enrollment process for chronic disease management, OSF HealthCare developed algorithms, validated internally by OSF OnCall, to help identify patients’ relevant chronic conditions and quantify overall clinical complexity and disease severity. It considers factors such as the number of comorbidities and hospitalizations in the past year. A score is generated that guides placement into one of three programs: OnCall Advanced Care (OCAC), Remote Patient Monitoring (RPM), or the Health and Wellness Program. Using this algorithm, patients with a qualifying risk score and a diagnosis of asthma, hypertension (HTN), diabetes mellitus (DM), heart failure (HF), or chronic obstructive pulmonary disease (COPD) are identified. Staff then conduct outreach to these patients to offer program enrollment.

##### 2.2.1.1 Advanced care and chronic disease management (OCAC—high touch)

The OCAC program aims to help patients manage their HTN, DM, HF, COPD, and asthma. Eligible patients are screened by program staff for suitability for the program (e.g., do not have a current substance abuse problem). Those selected are then contacted by program staff and enrolled in the program. Upon enrollment, a DHN assists patients in using a comprehensive care kit that includes a tablet and relevant home devices. Bluetooth peripheral devices send data into the EHR using a third-party vendor interface system. The OnCall staff respond to alerts triggered by patient responses to daily check-in questions and vital signs 24/7/365. Additionally, nurses contact patients approximately twice a week to check in, provide education, and help coordinate care with other healthcare providers. Depending on patient needs, patients also receive calls from pharmacists, dieticians, mental health providers, and social workers. In addition, patients are encouraged to message their care team through their tablet or call with any questions or concerns.

##### 2.2.1.2 Remote patient monitoring (RPM—medium touch)

A vendor-provided digital platform also facilitates outreach to patients eligible for the RPM program. If they choose to enroll in the program, they will have access to digital applications designed to help manage their health, wellness, and chronic diseases. Additionally, depending on their needs, they will receive home devices such as a scale, blood pressure cuff, blood glucose monitor, and peak flow meter. Communication primarily occurs through automated SMS text messaging and interactive voice response calls, which provide education and prompt patients to respond to health questions and report selected vitals. Depending on the patient's response, the RPM platform tailors the appropriate SMS text messaging or phone call response. An alert that triggers a call from a nurse or APP is fired when a patient reports a concerning symptom (such as increased swelling, weight gain, worsening shortness of breath) or enters a vital sign that falls outside the parameters of their set prescription. The RPM program monitors patients 24/7. Patients can initiate virtual visits with a nurse daily from 8 a.m. to 6 p.m. if needed. Symptom-based visits with a remote APP can be scheduled 24/7. Furthermore, health coaches provide help with virtual health and wellness at convenient times for patients.

##### 2.2.1.3 Health and wellness (low touch)

The Health and Wellness program aims to provide patients with educational resources about healthy living. A vendor-sourced platform is used to manage patients. As part of the program, patients have access to a digital Chatbot that they can use to chat with a triage nurse about medical questions. Additionally, OnCall nurses are available 24/7 to provide guidance on health-related issues, including questions about clinical conditions. Patients receive a weekly text message or phone call asking about their health/wellness journey to help them remain active in the program. Patients can contact a DHN anytime during the program for answers to questions and concerns. DHNs work with patients to coordinate communication with program staff at times that are convenient and selected by the patient. In 2024, a dietician was added as an additional resource for enrolled patients. Patients can also choose from various paths designed to enhance healthy behaviors (smoking cessation, exercise, diet, nutrition, and weight management).

##### 2.2.1.4 Pregnancy and postpartum support program (PPSP)

Obstetrics units are increasingly closing, leading to maternity deserts across the U.S. (19). Digital applications can be a resource for pregnant mothers by providing education and support throughout the pregnancy and postpartum period (20). Our PPSP program delivers education and support to mothers 24/7/365 through a vendor-based customizable digital care management program that can be downloaded to a smartphone or accessed via a computer. In addition, women who are at risk for or have hypertension are mailed a blood pressure cuff that is paid for by Medicaid. Upon enrollment, women are also screened for depression and are referred to resources as needed. Through the app, they receive education and weekly check-in questions tailored to where a mother is in her pregnancy or postpartum journey. Questions assess common pregnancy issues and self-reported data such as blood pressure readings. Concerning responses, activate digital alerts, which are sent to nurses who can contact the patients to perform triage or escalate care to APPs for a virtual visit. Chat functionality also allows a mother to communicate with the OnCall healthcare team to ask questions and express concerns. Program enrollees can also receive virtual lactation visits to facilitate breastfeeding.

### 2.3 Data infrastructure build with technology platforms

#### 2.3.1 Data infrastructure

The concept was to establish a modern platform and to adopt a data-as-a-product approach for managing and utilizing data across the ecosystem. To achieve this, we employed a data orchestrator platform [OSF Community Connect (OCC)], which supports the evolving requirements for enrolling in various programs. Associated barriers to its use by FQHCs included the need to address security compliance and to have complete segregation of data access. To ensure secure access and meet the privacy needs of various FQHCs, specific role-authorization protections have been implemented to restrict user access. OSF's data sources are stored within an Enterprise Data Warehouse (EDW), which serves as a centralized repository for integrated health data, managed by the data analytics division. Creating a digital data hub enables the sharing of health data from multiple sources. It encompasses functionalities such as discovering health datasets, providing metadata services, and ensuring data accessibility while adhering to regulations (21).

#### 2.3.2 Digital hub progression

The evolution of the digital hub has been ongoing, focusing on progress rather than achieving perfection. A technology, data, and reporting subcommittee was established to enhance collaboration and streamline processes (16). Third-party digital solutions often operate independently, necessitating careful integration to prioritize the patient's experience. The constant updates and adjustments in systems, such as the criteria for remote patient monitoring, changes in care escalation/de-escalation, and program participation, require different configurations. For instance, during the initial stages, patient enrollment and reconciling duplicate enrollment of the same patient from different sources required meticulous planning to manage multiple users’ experience journeys effectively. Understanding the user experience journey along a longitudinal path demanded careful consideration of failure modes. Thorough testing to identify and address potential failure points has been used to ensure program functionality with business, operational, and research team members engaged in the build/design meetings (16). Recently, as FQHCs have completed initial implementations, they have found value in being able to track outreach and enrollment in real-time for patients in the MIC Programs using the OCC platform's self-service functionality.

### 2.4 Pragmatic program evaluation and research

Program evaluation was incorporated into the design from the outset, specifically for OCAC, RPM, and PPSP using a pragmatic research approach. Klein et al. (16) provide further information about program evaluation processes, research conceptual framework, data and technology considerations, data curation and reliability challenges. A patient-centered research analysis is underway for the examination of OCAC and RPM health outcomes, patient engagement, and program usage using an observational model cohort design and qualitative interviews (see information available at ClinicalTrials.gov NCT05555095). Program feasibility and associations with maternal and infant health for the first year of the PPSP program are described elsewhere (22, 23).

## 3 Results

Gathering data is necessary to track the reach and adoption of these programs. Using Microsoft Power BI (24), program-specific dashboards were created that imported data from vendors and patients' EHRs. These dashboards were designed to track monthly enrollment metrics, patient engagement, and satisfaction by utilizing descriptive statistics. Thus, operational leaders can access this information in real time. The early success of this initiative is the satisfactory progress and completion of state-required milestones in the first 2 years of operation. Dashboard performance measures are described below.

### 3.1 Enrollment metrics

Since programs were implemented incrementally, outreach, enrollment, and select screening results were collected and reported to stakeholders (executive and operational leaders, care teams, FQHCS, and the state) monthly. In the 2 years since the launch of MIC, the number of patients who have been outreached for engagement in any one of the described programs is 418,037. Table 1 shows the quarterly enrollment volumes for unique new participants in the 2-year period (2022–2024), giving perspective for those interested in replicating this work. Vendor changes and technology builds occasionally impacted the capture of accurate numbers for enrollment or discharges from programs. Initially, some programs manually tracked enrollment. Most enrollment numbers have remained steady, with Health and Wellness showing an upward trend. Overall enrollment was higher than the prior year, with the average percentage of enrollment to outreach higher in the OCAC and PPSP programs.

**Table 1:** Quarterly program enrollment and screening metrics, September 2022 through August 2024^a^.

Program metrics	Sep.–Nov. 2022	Dec.–Feb. 2023	Mar.–May 2023	Jun.–Aug. 2023	Enroll-ment	Outreach Total	Sep.–Nov. 2023	Dec.–Feb. 2024	Mar.–May 2024	Jun.–Aug. 2024	Enroll-ment	Outreach Total	Average % Enrolled to Outreach
Enrollment													
Advanced care	56	92	52	28	**228**	422	71	43	56	80	**250**	1,541	24.4
Remote patient monitoring	1,349	400	825	466	**3,040**	31,192	1,021	360	1,094	480	**2,955**	25,037	11.8
Health & wellness	3,674	6,161	380	912	**11,127**	119,719	4,384	5,039	4,098	4,068	**17,589**	229,649	8.2
Pregnancy/Postpartum	590	381	389	432	**1,792**	4,541	613	369	588	413	**1,983**	5,936	36.0
Total	5,669	7,034	1,646	1,838	**16,187**	**155,874**	6,089	5,811	5,836	5,041	**22,777**	**262,163**	
Overall totals for 24-month time period:											**38,964**	**418,037**	

Bolded numbers reflect Enrollment subtotals within programs by year and overall total. Outreach total and Overall Outreach are also bolded.

^a^ Enrollment figures are provided for the first 24 full months after all programs started. Outreach total is for the same period. All numbers are unique new patients. Source: Authors.

### 3.2 Patient satisfaction and engagement metrics

#### 3.2.1 Patient satisfaction

In healthcare, many organizations have begun using the Net Promoter Score as a measure of patient satisfaction (25). Responses are categorized into three rating groups, ranging from 0 to 10 (or a similar scale), with 10 representing the most favorable: Promoters (9–10), Passives (7–8), and Detractors (0–6). Scores are based on a calculated percentage when considering the difference between promoters and detractors (26). The use of the NPS question of “likelihood to recommend” (LTR) is noted as beneficial for tracking longitudinal performance (26), which, at this time, serves as our primary measure of patient satisfaction. Additionally, others have noted that the LTR may be associated with the quality of care provided (27). Across these programs, NPS is calculated monthly and is available within the program-specific dashboard created using Power BI. Overall, program satisfaction has remained stable or trended positively from September 2023 to August 2024 (Table 2).

**Table 2:** Engagement and patient experience by program September 2023 to August 2024^a^.

Program metrics	Sept 2023	Oct 2023	Nov 2023	Dec 2023	Jan 2024	Feb 2024	Mar 2024	Apr 2024	May 2024	Jun 2024	Jul 2024	Aug 2024
Advanced care (OCAC)^b^												
Net promotor score	69.2	65.0	76.9	90	64.7	84.0	44.8	86.4	100	94.9	100	52.9
Remote patient monitoring (RPM)												
Engagement (%)	36.4	35.9	35.4	36.2	38.4	39.4	44.8	51.5	53.9	55.7	59.9	62.8
Net promotor score	66.6	66.7	76.6	63.8	66.3	80.9	71.5	61.2	75.5	69.7	78.8	80.0
Health & wellness												
Engagement (%)	18.1	17.5	17.7	17.9	20	18.3	22.1	28.8	29.6	32.2	32.9	34.5
Net promotor score^c^	-	-	-	-	-	27.6	56.4	39.6	45.6	34.8	37.9	63.2
Pregnancy/Postpartum (PPSP)												
Engagement (%)	75.2	74.4	68.5	73.6	70.2	71.1	75.1	76.9	81.0	79.9	83.1	81.7
Net promotor score	85.1	80.4	75.5	80.4	74.6	76.3	84.6	85.4	85.5	65	77.3	66.7

^a^ Within the OSF HealthCare programs of RPM, Health and Wellness and PPSP, engagement is defined as “responding to a text message, check-in question, or through messaging program staff weekly. Patients who responded in that week are considered 100% engaged.” The denominator for the engagement rate is the count of weeks for which the patient was actively enrolled in a program. Sample size is not reported in this table because program enrollment varies daily.

^b^ Engagement is not reported for the Advanced Care program on the internal dashboard.

^c^ Net promotor score (NPS) is calculated based on patient surveys; in months when the number of reported responses are 10 or less, NPS is not reported (-). Source: OSF HealthCare Power BI Dashboard.

#### 3.2.2 Patient engagement

Patient engagement is measured within several programs (Table 2); however, because different vendors support these programs, definitions and calculations of engagement vary, with metrics not always available to OSF leaders for informed decisions about program delivery. Therefore, an internal OSF metric was created, which defines engagement as “responding to a text message, check-in question, or through messaging staff weekly. Patients who responded in that week are considered 100% engaged.” Engagement is then calculated by the number of weeks a person is 100% engaged, divided by the number of weeks they are active in the program. The engagement metrics are available to program staff on the internal dashboard and are reported monthly in aggregate, excluding OCAC, as the care team is involved with these patients weekly due to their higher complexity. Because programs are designed differently, comparison of engagement between programs is not performed. Monthly percentages for engagement are higher in the PPSP program, with positive upward trends noted in the RPM and wellness programs.

Given these reported metrics, we have demonstrated the feasibility of outreach to the Medicaid population on a larger scale with patient-centered approaches to its delivery. Further exploration of engagement data for all programs is needed at the subpopulation level as other researchers have noted declines in program enrollment over time in socially disadvantaged populations (28, 29).

### 3.3 SDoH metrics

Patients were screened across seven SDoH domains: stress, social integration, financial strain, food security, housing stability, transportation, and safety (18). The top areas of need were identified based on patient responses to SDoH standardized screening questions. For each domain, patients indicated whether they had a need and whether they desired assistance. The data in Supplementary Figure S2 reflect 1 month of screening (2023), during which stress (50.8%), social integration (34.8%), and financial strain (30.5%) emerged as the most reported needs. While this report represents a single month, trends across multiple months have shown that these domains consistently rank among the top areas of need, alongside housing, although exact percentages vary.

## 4 Challenges and facilitators to implementation and evaluation

As with any new digital service in healthcare, there are obstacles to overcome. These challenges centered on screening for SDoH, gaining clinician buy-in, the effective use of technology by patients, and the collection and reporting of metrics. Below, we describe the specific issues and actions we took to overcome these challenges.

### 4.1 SDoH screening and reporting challenges

The first years of operation highlighted several issues related to SDoH screening and referral tracking. For instance, in the OCAC program, screenings are conducted by phone, while in the RPM and Health and Wellness programs, patients must “opt in” rather than “opt out” for SDoH questions which might have contributed to lower participation rates. Also, data interoperability limitations have, so far, prevented us from using alternative screening methods (e.g., text, email) or from effectively tracking referral outcomes.

Significant enhancements to data and processes were made to address these issues. These included EHR integration with adoption of SDoH questions (30), including functionality for text or email responses to questions. Importantly, closed-loop functionality to support patient connection with community-based organizations and to monitor resource obtainment was implemented in late 2024, with further refinement and expansion planned in 2025. In parallel, an SDoH dashboard created in Power BI gives better data accessibility and visibility for aggregated patient responses and trends.

### 4.2 Clinician buy-in

Clinician engagement of those not involved in digital programs (e.g., primary care physicians) in the early stages was critical for understanding programmatic functions. Specifically, we needed the clinicians to understand that the program is a new way to engage the patients with supplemental care and support between clinic appointments 24/7/365. To reinforce this concept with patients, the program teams stress the continued importance of in-person visits with their current providers during digital engagements. Additionally, the data and clinical information from the MIC programs must be readily available for non-MIC clinicians to review so they feel connected to the care that patients receive through digital services. Information is available through an EHR link (real-time access to patient health information); however, the added clinician time needed for this review may be a barrier for clinician use of this information. Following the care standards within organizations is also critical to ensure alignment of patient care across all entry venues, with awareness of state licensure limitations for practitioners. With an understanding of program goals, and how clinical care is escalated, clinicians were more likely to promote the MIC programs. Anecdotally, for some clinicians, digital care has reduced the time constraints with educational needs during in-person visits; therefore, it was seen as a benefit for patients and the practice setting.

### 4.3 Patient use and comfort with technology

As noted earlier, the patient population is diverse and resides in various rural and urban areas, thus having differing levels of access to, experience with, and comfort using technology. For some patients, technological support extends beyond blood pressure or glucometer measures, they also need to know how to enter their values, troubleshoot Bluetooth connectivity issues, and how to use a tablet (provided to those who need it), computer, or smartphone (depending on the program). We have addressed these issues by sending DHNs to patients' homes to train and support technology use, returning as often as necessary. Other issues in the first year of implementation were that the scales provided could not accommodate some patients' weight and others had difficulty using the blood pressure cuffs (or the cuffs were sized incorrectly).

### 4.4 Challenges of metric reporting

Variation in NPS scores within and between programs is evident, with some of the variation potentially attributable to fewer surveys completed in a month, thus higher response rates are needed for meaningful interpretation. Use of only vendor-reported patient satisfaction metrics was a starting point given the priority focus on access, engagement, data infrastructure, and real-world application of digital healthcare in the public health population (4, 31). Use of multiple vendors also contributes to challenges. Therefore, we designed entry and exit surveys tailored to each digital program and delivered them to patients via secure text messaging at predefined points in their journey using the OCC platform. However, the survey build was not completed until after the initial programs' implementation. As others have also noted (32), the integration of numerous data sources has been complicated, requiring added time for testing and ensuring patient data security. The barrier has been the time delays associated with creating an automated platform and delivery to patients, which pushed out the final activation of these entry/exit evaluation surveys to October 2024. A key lesson learned from a preliminary review of this survey data is the importance of meticulously mapping and orchestrating processes, considering various points of program entry and exit to minimize confusion.

## 5 Current state of program implementation and evaluation

### 5.1 Staffing and changes to staffing after implementation

Various digital teams are continuing to work towards optimization and efficiency, engaging clinical and operational leaders as needed. Care models and staffing needs have been evaluated and revised over time as the programs expanded to the FQHCs. As an example, the OCAC program implemented a new care model in January 2024, which consists of two RNs monitoring disease-specific patients, one RN performing scheduled visits, and one medical assistant (MA) focusing on trends and outreach calls. This has streamlined and ensured the proper use of skill sets for both roles. The MA calls each patient on their first day of enrollment, which helps foster an understanding of the program. As of February 2024, 179 nurse visits were completed for the month, with 1,929 active patients. The MA has completed 2,456 phone calls and two-way messages in 2 months. In the second year, 7.3 new positions were added, and a staffing model with medical assistants was implemented for the RPM program.

### 5.2 Measuring and tracking patient outcomes

In Year 3, dashboards for real-time metric monitoring remain part of the optimization planning and evaluation. Tracking and evaluation of patient outcomes, including hospitalizations (emergency, inpatient, and observation stays), hospital readmissions, patient self-reported blood pressures, and hemoglobin A1C results, are currently in pilot testing and development, along with dashboards to monitor digital interactions (virtual visits, clinical alert responses, and resolutions). As noted earlier, the use of multiple external vendors have impacted metrics reporting and measurement. Additionally, not all patients are affiliated with OSF HealthCare, so patient data may be stored in various locations. As a result, identifying and validating data, as well as evaluating health outcomes, has been difficult. We continue to collaborate with IT, data analytics teams, vendors, and clinicians to identify data sources and assess data quality. We have finalized the data use agreement with the state and are now examining data-sharing processes for medical and claims data when patients seek care outside our collaborative.

## 6 Discussion

### 6.1 Implications for collaborative partnerships

Since the start of the COVID-19 pandemic, there has been rising interest in utilizing telehealth as a method to improve disease prevention and management, as well as to increase access to prenatal care. Our experience in creating and implementing these programs has demonstrated that they can reach more patients and potentially impact our communities' health more than relying solely on in-person clinics. The growth in this field has largely been driven by changes in reimbursement from public and private insurance, which have aided in the development and funding of these programs (33). While we were able to expand our programs with grant support from Illinois, the significant effort required to implement these programs, coupled with the uncertainty surrounding future coverage for this type of care, necessitates policy changes to establish standard payment models that incorporate payment and coverage parity (34) to support their development in other locations and internationally (35).

Our recommendations and observations below are provided for public health, community, and health system leaders who are interested in replicating this work:

• Innovation focuses on developing novel strategies; we suggest supporting teams to live with ambiguity during implementation and to adapt to circumstances and unforeseen challenges.
• Devote time and effort into helping FQHC partners understand the key components of digital health. Patient journeys can help partner organizations to see the ways care can be delivered through technology.
• Seek to develop solid relationships between collaborative partners through a series of working meetings over the course of the collaborative effort.
• Consider building a quality structure similar to a hospital entity including a quality committee, peer review, ongoing and focused professional peer evaluation.
• With data that supports digital program need in communities, find ways to engage payors for funding support.

Our future steps include use of FQHC associated data and state reported data to better understand how the MIC digital programs could support patient care. Data use agreements that respect patient privacy and restrictions will likely be necessary and helpful for program evaluation of health-related outcomes.

## 7 Conclusion

The MIC program provides some insights into how state/private/public partnerships can support evidence-based digital health programs that can be used to manage pregnancy and chronic diseases. In time, the MIC program evaluation could provide the basis for the continued expansion of telehealth services. While we are not yet able to report on the programs' impact on the outcomes of chronic diseases, early results show favorable levels of patient satisfaction and demonstrate promise for program sustainability over the next several years and beyond. Bold, innovative strategies that leverage community partnerships are needed in today's world as we grapple with the limited number of available resources. We believe that one or more of our programs could be adopted by others seeking to better meet patients' needs.

## Data Availability

The datasets presented in this article are not readily available because the data that are described in this article are provided in aggregate and disclosed in accordance with the MIC Collaborative agreement, which restricts the sharing of data at the individual level. Data are also protected and restricted by the Institutional Review Board approval of research protocols of the MIC research team. Requests to access the datasets should be directed to melinda.b.cooling@osfhealthcare.org.
