# Supplementary material for: Increasing access to care through digital health for the Medicaid population: a novel community case study
Source: Front Digit Health. 2025 Sep 19;7:1524590. doi: 10.3389/fdgth.2025.1524590 (PMC12492634; doi:10.3389/fdgth.2025.1524590)
Supplement: Supplementary file 1 [file Datasheet1.pdf]

| Milestones and Deliverables for MIC – Excerpt | Calendar Year 1 (Feb 22 – Dec 2022) | | | | Calendar Year 2 (Jan 23 – Dec 2023) | | | |
|---|---|---|---|---|---|---|---|---|
| **Quarterly by Year for Contract Implementation - Beginning Feb 1, 2022** | **Q1** | **Q2** | **Q3** | **Q4** | **Q1** | **Q2** | **Q3** | **Q4** |
| | | | | | | | | |
| **Collaborative Partnership Milestones** | | | | | | | | |
| Create Governance Committee (pre-implementation - foundational) | | | | | | | | |
| Communication and orientation to program including measurable goals | | | | | | | | |
| Federally Qualified Health Center (FQHC) Understanding/Integration of OSF OnCall Community Connect (OCC) Programs and Promotion of OnCall with FQHC patients | | | | | | | | |
| Begin to evaluate future needs for Social Determinants of Health (SDoH) resources | | | | | | | | |
| Create plan to enhance programs based on patient and community partner feedback | | | | | | | | |
| Increase enrollment for digital health OnCall programs with proven outcomes by 5-20% depending program and entity. (Evaluated monthly and reported annually) | | | | | | | | |
| **OSF OnCall Program Milestones** | | | | | | | | |
| Design health literacy/wellness program | | | | | | | | |
| Launch digital outreach for mammography screening-1st batch and ongoing throughout program (program details not described in manuscript) | | | | | | | | |
| Begin OSF patient enrollment in established OnCall programs (OCAC) | | | | | | | | |
| Begin FQHC patient enrollment in established OnCall programs (FQHC #1-4) | | 1 | | 2 | 3 | 4 | | |
| Design Pregnancy & Postpartum Program (PPSP) and enroll FQHC patients (FQHC #1-4) | | | 1, 2 | 3 | 4 | | | |
| Hire simulation program development specialist to begin Community Health Workers (CHW), Digital Health Navigators (DHN) curriculum design (program details not described in manuscript) | | | | | | | | |
| Deploy digitally enabled CHWs/DHNs to site-specific locations | | | | | | | | |
| Create plan to enhance programs based on patient and community partner feedback | | | | | | | | |
| Design behavioral health program milestone in Years 3 – 4 (program details not described in manuscript) | | | | | | | | |
| **OSF OnCall Data/Technology Milestones** | | | | | | | | |
| Optimize and enhance OCC technology platform | | | | | | | | |
| Deploy and integrate OCC technology platform into FQHC (1-3) | | | | 1 | 2 | 3 | | |
| Begin to utilize predictive modeling for targeted populations | | | | | | | | |
| FQHC EMR Assessment | | | | | | | | |
| FQHC Begin EMR Install for compatible EMR (Epic) | | | | | | | | |
| FQHC Education and Training on EMR | | | | | | | | |
| FQHC “go live” for implementation | | | | | | | | |
| Optimize and enhance technology platform for data sharing | | | | | | | | |

**Supplementary Figure 1.** Excerpt of Select Key Milestones and Deliverables for OSF OnCall Medicaid Innovation Collaborative. ©2024, OSF HealthCare.

*Legend:* Light blue = goal to start; Yellow = in progress; Dark Blue = completed.
OCAC= OnCall Advanced Care Program. 1=FQHC #1; 2= FQHC 2; 3= FQHC 3; 4= FQHC 4.

Adapted from OSF HealthCare Transformation Program Medicaid Innovation Collaborative Agreement. Used with permission.
