# Supplementary material for: Increasing access to care through digital health for the Medicaid population: a novel community case study
Source: Front Digit Health. 2025 Sep 19;7:1524590. doi: 10.3389/fdgth.2025.1524590 (PMC12492634; doi:10.3389/fdgth.2025.1524590)
Supplement: Supplementary file 2 [file Datasheet2.pdf]

Supplementary Figure 2. Social Determinants of Health (SDoH) Screening – Excerpt of Monthly Data for 1 Month in 2023**

STRESS

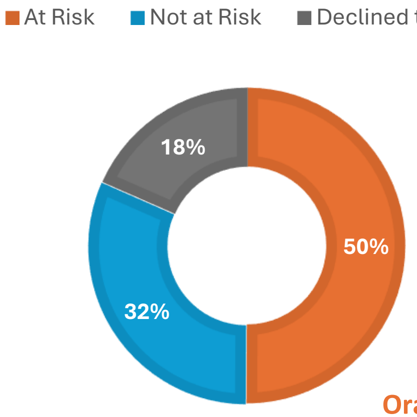

Orange - At risk = 615
Blue - Not at risk = 387
Grey - Declined to answer = 226

FINANCIAL RESOURCES STRAIN

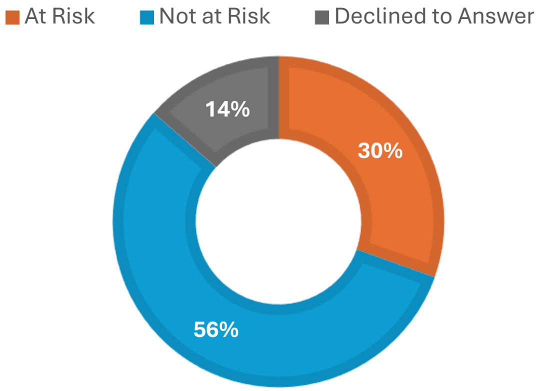

Orange - At risk = 374
Blue - Not at risk = 687
Grey - Declined to answer = 167

FOOD INSECURITY

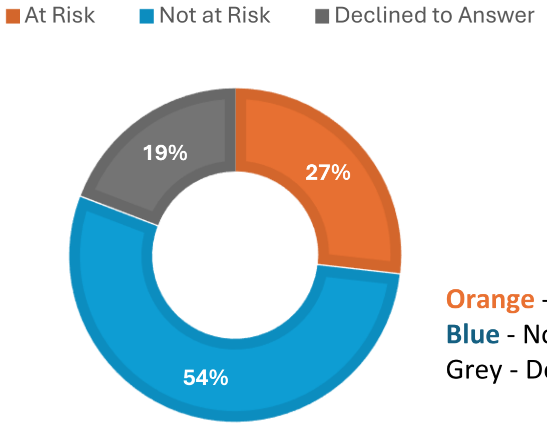

Orange - At risk = 339
Blue - Not at risk = 663
Grey - Declined to answer = 236

Overall Screenings
1228

Resources Provided[a]
154

Race/Ethnicity	
American Indian or Alaska Native	*
Asian	*
Black or African American	229
Native Hawaiian	*
Other or Unknown	102
Other Pacific Islander	*
White or Caucasian	872
Hispanic	81

Gender	
Female	798
Male	430

Excerpt of monthly data reported to the state for the Medicaid Innovation Collaborative (MIC). SDoH screenings were tracked by OSF Medical Group adult primary care practices outside of the MIC and by OSF OnCall in support of the MIC.

[a] Resources provided identifies the number of recommendations and/or resources offered for the domains for which they screen positive AND for which they have indicated they want assistance.

Percentages for SDoH reflect responses or "declined to answer" for each specific domain of need. Patients may exhibit more than one area for needs or race/ethnicity, thus may be represented in more than 1 domain.

*Number of respondents is less than 20 - Exact numbers not provided to ensure privacy
** Exact month not provided to ensure privacy

Source: Authors. © OSF HealthCare.
